# Neonatal abstinence syndrome hospitalizations in Canada: a descriptive study

**DOI:** 10.17269/s41997-022-00726-5

**Published:** 2022-12-08

**Authors:** Rebecca Plouffe, Vera Grywacheski, Wei Luo, Chantal Nelson, Heather Orpana

**Affiliations:** 1 Centre for Surveillance and Applied Research, Health Promotion and Chronic Disease Prevention Branch, Public Health Agency of Canada, Ottawa, Ontario Canada; 2 School of Epidemiology and Public Health, University of Ottawa, Ottawa, Ontario Canada

**Keywords:** Neonatal abstinence syndrome, Canada, Opioids, Maternal substance use, Pregnancy, Neonatal withdrawal, Syndrome d’abstinence néonatale, Canada, opioïdes, consommation de substances par la mère, grossesse, sevrage néonatal

## Abstract

**Objective:**

The objective of this paper is to describe the trend of newborn hospitalizations with neonatal abstinence syndrome (NAS) in Canada, between 2010 and 2020, and to examine severity indicators for these hospitalizations.

**Methods:**

National hospitalization data (excluding Quebec) from the Canadian Institute for Health Information’s Discharge Abstract Database, from January 2010 to March 2021, and Statistics Canada’s Vital Statistics Birth Database were used. Analyses were performed to examine NAS hospitalizations by year and quarter, and by severity indicators of length of stay, Special Care Unit admission and status upon discharge. Severity indicators were further stratified by gestational age at birth.

**Results:**

An increasing number and rate of NAS hospitalizations in Canada between 2010 (*n* = 1013, 3.5 per 1000 live births) and 2020 (*n* = 1755, 6.3 per 1000 live births) were identified. A seasonal pattern was observed, where rates of NAS were lowest from April to June and highest from October to March. Mean length of stay in acute inpatient care was approximately 15 days and 71% of NAS hospitalizations were admitted to the Special Care Unit. Hospitalizations for pre-term births with NAS had longer durations and greater rates of Special Care Unit admissions compared to term births with NAS.

**Conclusion:**

The number and rate of NAS hospitalizations in Canada increased during the study, and some infants required a significant amount of specialized healthcare. Additional research is required to determine what supports and education for pregnant people can reduce the incidence of NAS hospitalizations.

## Résumé

**Objectif:**

Le présent article a pour but de décrire la tendance des hospitalisations de nouveau-nés atteints du syndrome d’abstinence néonatale (SAN) au Canada, entre 2010 et 2020, et d’examiner les indicateurs de gravité de ces hospitalisations.

**Méthode:**

Les données nationales sur les hospitalisations (à l’exclusion du Québec) provenant de la base de données sur les congés des patients de l’Institut canadien d’information sur la santé, de janvier 2010 à mars 2021, ainsi que la base de données sur les naissances des statistiques de l’état civil de Statistique Canada ont été utilisées. Des analyses ont été réalisées pour examiner les hospitalisations liées au SAN par année et par trimestre, et par indicateurs de gravité de la durée du séjour, de l’admission dans une unité de soins spéciaux et de l’état à la sortie de l’hôpital. Les indicateurs de gravité ont en outre été stratifiés en fonction de l’âge gestationnel à la naissance.

**Résultats:**

Un nombre et un taux croissants d’hospitalisations liées au SAN au Canada entre 2010 (*n*=1 013, 3,5 pour 1 000 naissances vivantes) et 2020 (*n*=1 755, 6,3 pour 1 000 naissances vivantes) ont été identifiés. Une tendance saisonnière a été observée, où les taux de SAN étaient les plus bas d’avril à juin et les plus élevés d’octobre à mars. La durée moyenne du séjour en soins de courte durée était d’environ 15 jours et 71 % des hospitalisations liées au SAN ont été admises à l’unité de soins spéciaux. Les hospitalisations pour les accouchements prématurés de nouveau-nés atteints du SAN avaient des durées plus longues et des taux plus élevés d’admissions dans des unités de soins spéciaux par rapport aux naissances à terme de nouveau-nés atteints du SAN.

**Conclusion:**

Le nombre et le taux d’hospitalisations liées au SAN au Canada ont augmenté au cours de l’étude, et certains nourrissons nécessitent une quantité importante de soins spécialisés. Des recherches supplémentaires sont nécessaires pour déterminer quels soutiens et quelle éducation pour les personnes enceintes peuvent réduire l’incidence des hospitalisations liées au SAN.

## Introduction

Canada is currently experiencing an opioid crisis, affecting people across the country and across all ages and socioeconomic groups (Belzak and Halverson, 2018). Over the past decade, opioid-related harms, including hospitalizations and mortality, have increased drastically (Haas, 2019). A population particularly vulnerable to the harms associated with opioid use are pregnant people and their offspring (Haas, 2019). Fetuses exposed to certain substances during pregnancy can result in a set of withdrawal symptoms experienced by a newborn, referred to as neonatal abstinence syndrome (NAS) (Canadian Institute for Health Information, 2018). NAS most commonly occurs when a fetus is exposed to opioids during pregnancy, but can also occur through fetal exposure to antidepressants, benzodiazepines, nicotine, caffeine, alcohol, methamphetamine, and inhalants (Haas, 2019; Filteau et al., 2018). With respect to opioids, maternal use of stronger agonists (e.g., oxycodone, morphine) has been associated with a higher risk of NAS compared to weaker agonists (e.g., codeine, hydrocodone) (Esposito et al., 2022). It is difficult to obtain comprehensive data on prenatal opioid exposure. However, a population-based study in Ontario found that between 2006 and 2011, 67% of mothers on public drug plans who delivered an infant with NAS received an opioid prescription in the 100 days preceding delivery (Turner, 2015). Symptoms of NAS include irritability, poor feeding, tremors, diarrhea, respiratory distress, and seizures and typically present within 24–74 h of birth (Filteau et al., 2018; Lacaze-Masmonteil and O’Flaherty, 2018; McQueen and Murphy-Oikonen, 2016). NAS may also have long-term developmental consequences, including mental health and behavioural problems, poor school performance, motor deficits, and visual disorders (Rees et al., 2020; McQueen and Murphy-Oikonen, 2016; Logan et al., 2013). These associations remain unclear, however, due to the impact of other environmental factors such as socioeconomic status, parental education, nutrition, prenatal care, and postnatal environmental stability and are therefore difficult to measure and understand (McQueen and Murphy-Oikonen, 2016; Logan et al., 2013).

There is some evidence to suggest that rates of NAS have been increasing in Canada; however, there are limited published data for recent estimates as well as for examining rates of NAS in Canada over a longer period of time (Canadian Institute for Health Information, 2018; Lisonkova, 2019). NAS is also associated with significant healthcare costs, due to prolonged hospital stays, Special Care Unit admission, pharmacological treatments, and birth complications (Haas, 2019; Canadian Institute for Health Information, 2018; Lacaze-Masmonteil and O’Flaherty, 2018; McQueen and Murphy-Oikonen, 2016).

The objective of this analysis was to describe the trend of hospitalizations for newborns with NAS in Canada (excluding Quebec) between 2010 and 2020. Other indicators of severity were examined, including length of stay, Special Care Unit admission, and status upon discharge from hospital. These severity indicators were stratified by gestational age at birth (pre-term or term delivery). NAS hospitalizations were also analyzed to determine whether a seasonal trend existed. As seasonal trends have previously been identified with opioid use and associated harms, primarily finding increased opioid use and opioid-related hospitalizations in summer months (Stopka et al., 2021; Houser et al., 2021), it was of interest to determine whether seasonal trends exist among other substance-related harms as well. The results of this analysis can be used to inform future parental and child health practices and related harm-reduction strategies in Canada.

## Methods

### Data source

Hospitalization data for newborns were obtained from the Canadian Institute for Health Information’s (CIHI) Discharge Abstract Database. These data include acute inpatient hospital discharge records for all newborns hospitalized across Canada, excluding Quebec. This would include any newborn hospitalized, regardless of whether the pregnant parent was a Canadian resident or not. The number of live births by year, month, and mother’s place of residence was gathered from Statistics Canada’s Vital Statistics Birth Database (Statistics Canada, 2021a, 2021b). Data for the period from January 2010 to March 2021 were included in this study.

### Variables

The International Classification of Diseases and Related Health Problems, Tenth Revision, Canada (ICD-10-CA) was used to capture diagnoses from the newborn’s hospitalization. The methodology used to identify NAS hospitalizations was adapted from existing CIHI methodology (Canadian Institute for Health Information, 2018). NAS hospitalizations were identified as neonates who were born in hospital and received a significant diagnosis of NAS before discharge, or neonates admitted within 28 days of birth and received a significant diagnosis of NAS. This analysis was limited to live births only. These records were captured using the ICD-10-CA code “P96.1”, defined as “neonatal withdrawal symptoms from maternal use of drugs of addiction, includes neonatal abstinence syndrome. Excludes reactions and intoxications from maternal opiates and tranquillizers administered during labour and delivery”. Only significant diagnosis types, where NAS was influential to the time spent and treatment received in hospital, were included. Query or unconfirmed diagnoses of NAS and stillbirth records were excluded from analysis. Similar methods, including the use of the ICD-10-CA code P96.1, have also been used in Ontario, although our study applied additional criteria to exclude unconfirmed or query diagnoses and examined only significant diagnoses of NAS (Turner, 2015). For the stratification of births into term and pre-term, a pre-term birth was defined as liveborn infants with a gestational age at birth of less than 37 weeks old, whereas a term birth was defined as infants with a gestational age at birth of 37 weeks old and greater.

For measures of severity, the total length of stay in hospital was analyzed as the sum of the number of days the infant was in acute inpatient care and/or alternate level of care. Acute inpatient care length of stay describes when patients are receiving necessary treatment for a disease or severe episode of illness for a short period, whereas alternate level of care describes when patients are occupying a bed, but not requiring the intensity of services provided in that care setting (Canadian Institute for Health Information, n.d.-a, n.d.-b). Discharge disposition was explored and refers to the status of the patient upon discharge or where the patient is discharged to. The possible outcomes for discharge disposition include discharge home with additional support, discharge home without additional support, transferred to another hospital, left against medical advice, or died in hospital. Home without additional support is defined as being discharged to a private home, condominium, or apartment without supports from the community at home or referred to services. Parents or guardians of an infant in this group may have been provided instructions to return to their general practitioner or were referred to a specialist as part of a routine discharge order. Finally, Special Care Unit admission was analyzed as the proportion of hospitalizations where the patient was admitted to a Special Care Unit, such as the Neonatal Intensive Care Unit. All severity indicators were identified by examining mandatory fields within the newborn’s hospitalization abstract.

Seasons were defined in this analysis by their calendar quarter. Quarter 1 represented January–March, Quarter 2 April–June, Quarter 3 July–September, and Quarter 4 October–December. Seasons were defined in this way as they most accurately represent the four distinct seasons of the year in Canada and are how quarters are most commonly defined.

### Analyses

A descriptive analysis from 2010 to 2020, by year and by quarter, was completed for NAS hospitalizations in Canada; both counts and crude rates were reported. Crude rates of NAS hospitalizations per 1000 live births were calculated for each year and quarter. Live birth data for Quebec were excluded to match the coverage of available hospitalization data from the Discharge Abstract Database. In addition, as live birth data for Yukon were not consistently available for all data years, Yukon was excluded from reporting of crude rates, but included when presenting counts.

A trend analysis, including NAS hospitalization rates from 2010 to 2020, was examined using the X13 procedure in SAS (SAS Help Center, 2017). Quarterly rates of NAS hospitalizations were used in the automatic model selection procedure of the X13 ARIMA method to identify orders of differencing and the ARIMA model that best fits the data. This procedure identifies which model has the smallest BIC and BIC2 values compared to other models and checks for unit roots, overdifferencing, insignificant ARMA coefficients, and an adequate confidence coefficient of the Ljung-Box Q statistic. Using the X13 procedure, the data were tested for seasonality using the identified best fitting model. The X13 procedure tests for identifiable seasonality by combining *F*-tests for stable and moving seasonality and the Kruskal-Wallis test for stable seasonality.

Differences in both mean acute inpatient and alternate level of care length of stays in hospital from 2010 to 2020 were examined using a one-way repeated measures analysis of variance (ANOVA). Mean length of stay for both levels of care was calculated by quarter to be matched throughout the years. Additional severity indicators such as Special Care Unit admissions and status upon discharge were calculated using data from April 2019 to March 2020 and April 2020 to March 2021. Both years are presented in order to explore whether these hospitalization characteristics were impacted by the COVID-19 pandemic in the April 2020 to March 2021 data year. NAS hospitalizations and severity indicators were stratified by pre-term and term births using the April 2020 to March 2021 data year. *T*-tests and chi-square tests were completed to determine differences in severity indicators between pre-term and term births. Counts of five and less were suppressed in accordance with CIHI’s privacy policy. All analyses were completed using SAS Enterprise Guide version 7.1 and R version 4.1.1.

## Results

**Fig. 1:**
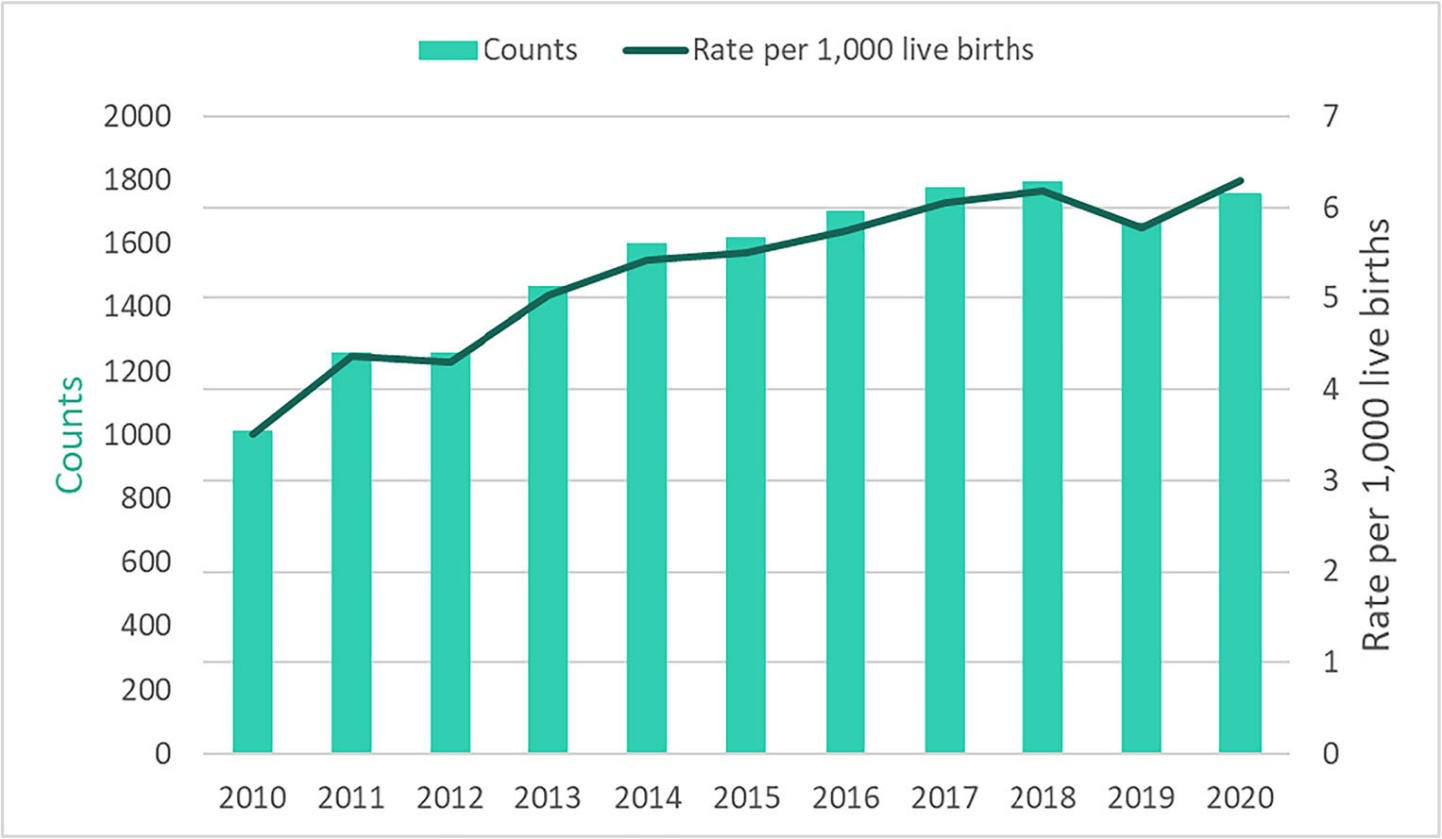
Counts and crude rates of hospitalizations for neonatal abstinence syndrome from 2010 to 2020, by calendar year

Across Canada (excluding Quebec) from 2010 to 2020, there were 16,920 NAS hospitalizations. Table 1 provides a demographic description of these hospitalizations. In 2020, there were 1755 NAS hospitalizations, representing a 73% increase in the number of hospitalizations from 2010 (*n* = 1013) and a 5% increase in the number of hospitalizations from 2019 (*n* = 1665) (Fig. 1). In terms of crude rates, in 2010 there were 3.5 NAS hospitalizations per 1000 live births, which increased by 80% to 6.3 per 1000 live births in 2020. Of note, there appeared to be a dip in the rate of NAS hospitalizations in 2019; however, the rate increased to the highest level recorded in 2020.

**Table 1:** Neonatal abstinence syndrome hospitalizations in Canada (excluding Quebec), demographic characteristics, 2010–2020

	**Neonatal abstinence syndrome hospitalizations**
	***N*** **= 16,920** **% (*n*)**
**Sex**	
Female	46% (7712)
Male	54% (9208)
Age at time of admission	
0 days	88% (14,858)
1–4 days	6% (1002)
5–9 days	3% (531)
≥ 10 days	3% (529)
Gestational age at birth	
< 22 weeks	0% (0)
22–27 weeks	1% (85)
28–31 weeks	2% (275)
32–33 weeks	3% (512)
34–36 weeks	18% (3034)
37–41 weeks	76% (12,824)
≥ 42 weeks	1% (190)
Weight (g)	
< 1000 g	0% (69)
1000–1499 g	1% (203)
1500–1999 g	4% (728)
2000–2499 g	14% (2379)
2500–2999 g	29% (4938)
3000–3499 g	31% (5325)
3500–3999 g	15% (2486)
4000–4499 g	4% (650)
≥ 4500 g	1% (135)
Missing	0% (7)
Low birth weight (≤ 2499 g)	
Yes	20% (3379)
No	80% (13,541)

**Fig. 2:**
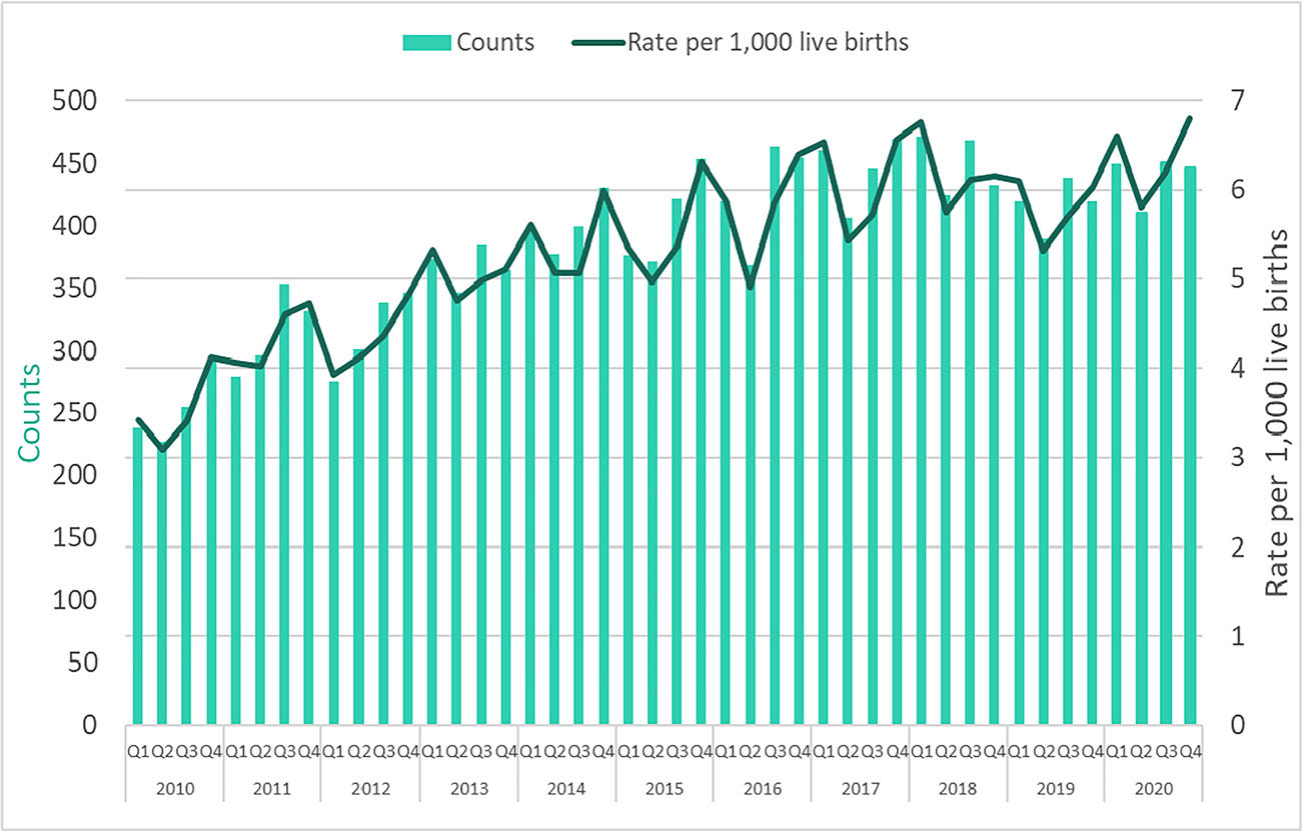
Counts and crude rates of hospitalizations for neonatal abstinence syndrome from 2010 to 2020, by quarter

**Fig. 3:**
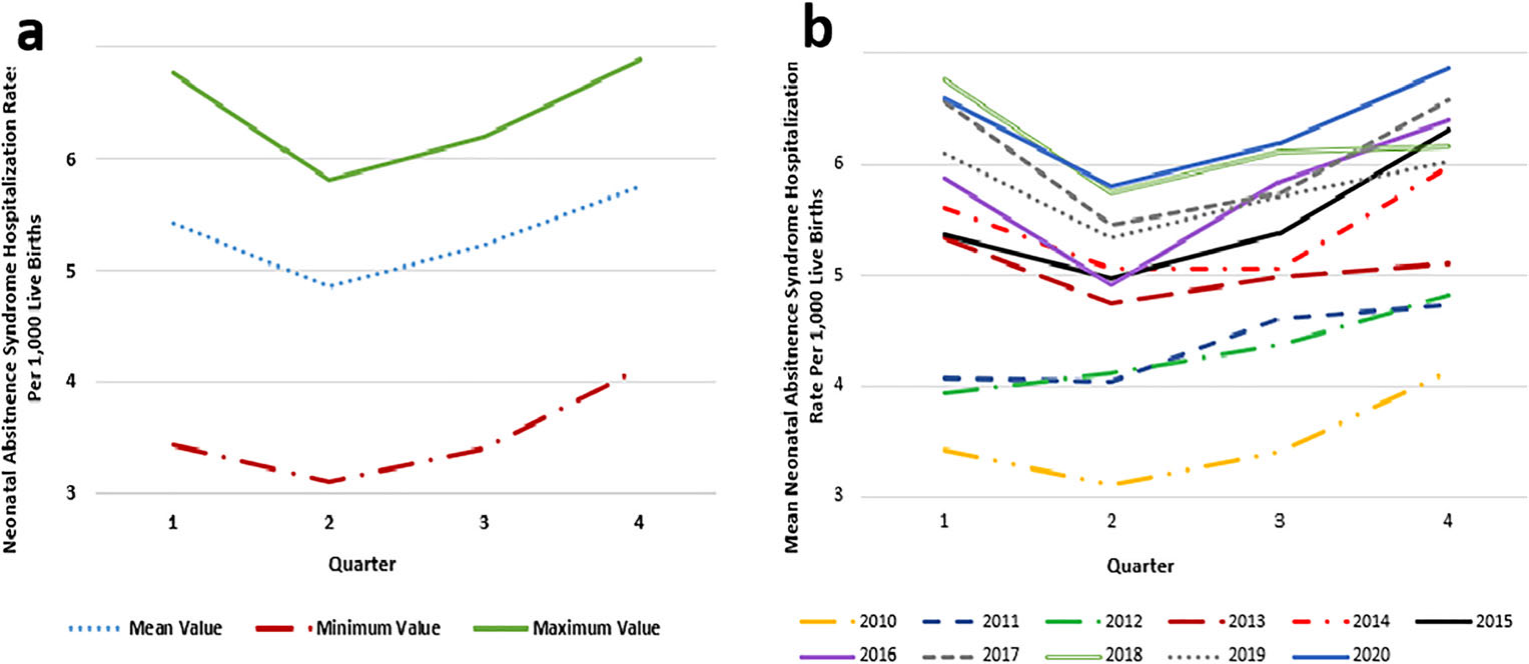
Seasonality of neonatal abstinence syndrome hospitalizations in Canada, excluding Quebec. **a** Mean, minimum, and maximum values of neonatal abstinence syndrome hospitalization rate, by quarter from 2010 to 2020. **b** Quarterly mean neonatal abstinence syndrome hospitalization rate, by year

NAS hospitalizations were examined by quarter as shown in Fig. 2. Results of a time series analysis supported a seasonality effect, with the mean NAS hospitalization rate being highest in quarter 4 (October to December, mean rate = 5.74) and lowest in quarter 2 (April to June, mean rate = 4.84) (Fig. 3a and b). Using the automatic model selection procedure, the (0,1,0) (0,1,1) model was chosen to best fit the NAS hospitalization rate data. These data indicate intrayear variation that is constantly repeated, as demonstrated by the stable seasonality test, *F* = 38.31, *p* < 0.001. Moving seasonality, indicating intrayear variation evolving from year to year, was not present, *F* = 0.929, *p* = 0.521, while nonparametric stable seasonality was present, Kruskal-Wallis chi-square = 31.274, *p* < 0.001. Overall, identifiable seasonality of NAS hospitalization rate is present and does not evolve year after year.

**Fig. 4:**
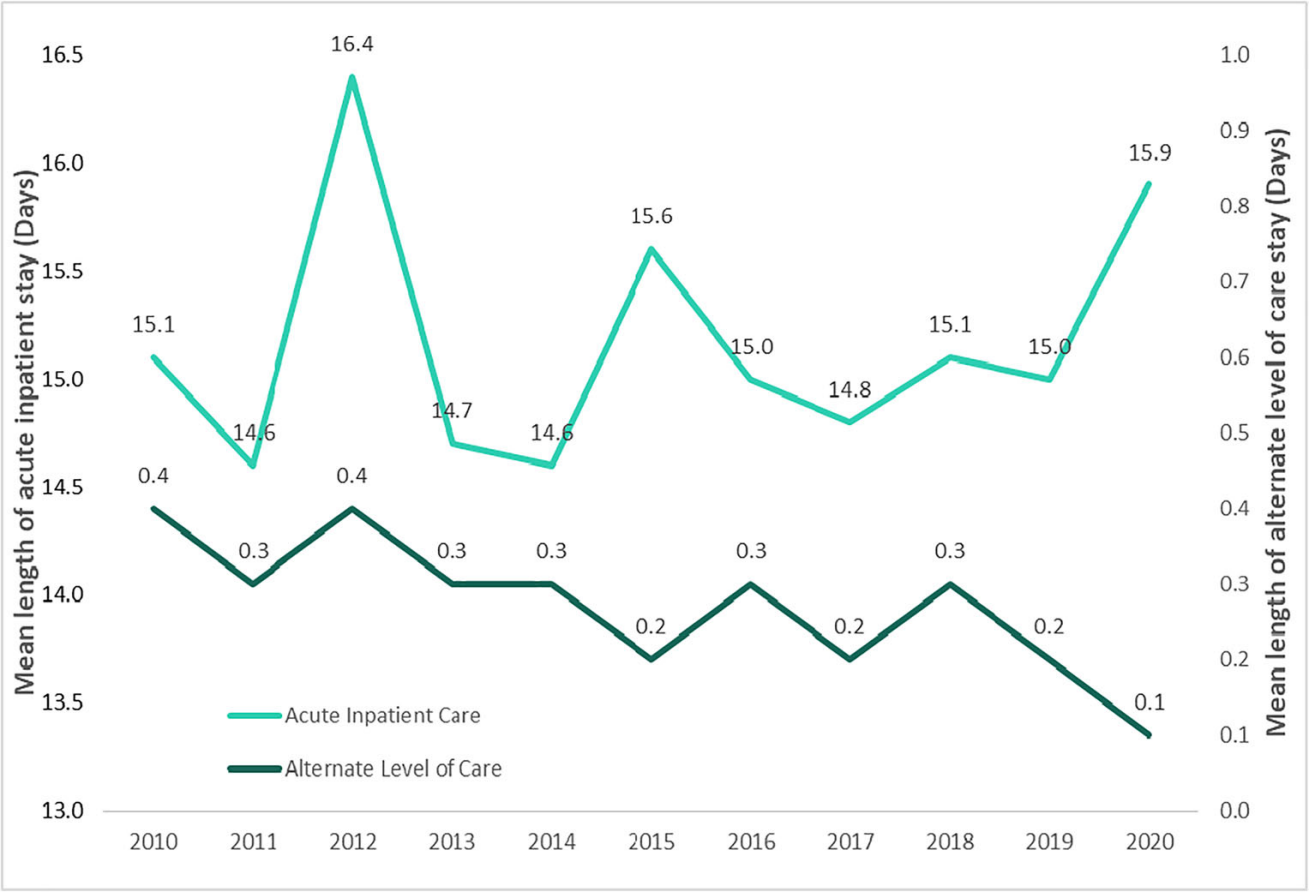
Mean length of stay in hospital for neonatal abstinence syndrome hospitalizations by calendar year, Canada (excluding Quebec), 2010–2020

Mean length of stay in acute inpatient care varied throughout the years, but was highest in 2012 at 16.4 days and lowest in 2011 and 2014 at 14.6 days (Fig. 4). Based on an ANOVA, no statistically significant difference was found in the mean length of stay in acute inpatient care among infants hospitalized with NAS, between 2010 and 2020, *F*(10, 30) = 1.435, *p* = 0.213 (Fig. 3). Mean length of stay in alternate level of care was highest in 2010 and 2012 at 0.4 days and appeared to gradually decrease while declining to its lowest in 2020 at 0.1 days. A statistically significant difference was identified in mean length of stay in alternate level of care over the same period, *F*(10,30) = 2.807, *p* < 0.014. Post hoc Bonferroni tests revealed that no pairwise comparisons were statistically significant. Additional severity indicators for NAS hospitalizations are presented in Table 2. From April 1, 2019 to March 31, 2020, the majority (71%) of NAS hospitalizations were admitted to the Special Care Unit to receive additional care. In terms of discharge disposition, most (72%) of NAS hospitalizations were discharged home without additional support, while 15% were discharged home with additional supports, for instance with a referral to additional services or with a home care program. A smaller percentage (12%) of neonatal abstinence syndrome hospitalizations resulted in the patient being transferred, for instance to another hospital for inpatient care, to group living, or to supportive housing. There were less than five deaths in hospital among newborns with NAS. Data from the most recent data year (April 1, 2020–March 31, 2021), a period impacted by the COVID-19 pandemic, indicated very similar findings. Among newborns hospitalized with NAS from April 2020 to March 2021, 71% were admitted to the Special Care Unit, 70% were discharged home without additional support, 16% were discharged home with additional support, and 13% were transferred.

**Table 2:** Neonatal abstinence syndrome hospitalizations, by severity indicators in Canada (excluding Quebec), April 2019-March 2020 and April 2020-March 2021

	**April 2019–March 2020**	**April 2020–March 2021**
	***N*** **= 1695** **% (*n*)**	***N*** **= 1723** **% (*n*)**
Special Care Unit admission	71% (1199)	71% (1216)
Discharge disposition		
Home with additional support	15% (260)	16% (273)
Home without additional support	72% (1226)	70% (1214)
Transferred	12% (188)	13% (232)
Left against medical advice, absent or did not return from pass	Suppr.	Suppr.
Died in hospital	Suppr.	Suppr.

*Suppr.*, data are suppressed in accordance with CIHI’s privacy policy

Severity indicators for NAS hospitalizations also varied by pre-term or term birth status (Table 3). In the most recent data year (April 1, 2020–March 31, 2021), 27% of hospitalizations for NAS were pre-term and 73% were term. In examining length of stay in hospital for NAS, the mean duration of acute care was longer for pre-term compared to term births (20.0 vs. 14.7 days), *t*(1) = 5.97, *p* < 0.0001. Additionally, a higher proportion of hospitalizations for pre-term births with NAS were admitted to the SCU compared to term births (89% vs. 64%), *X*^2^(1) = 105.45, *p* < 0.0001. Pre-term births also had a higher proportion of discharges home with additional support (20% vs. 14%), *X*^2^(1) = 10.15, *p* = 0.0014, and transfers (18% vs. 12%), *X*^2^(1) = 12.86, *p* = 0.0003, compared to infants with a term birth with NAS.

**Table 3:** Neonatal abstinence syndrome hospitalization severity indicators, by term or pre-term birth in Canada (excluding Quebec), April 2020–March 2021

	**Pre-term birth**	**Term birth**	***t*-statistic (df)**	***X***^**2**^ **statistic (df)**	***p*-value**
	**(< 37 weeks at delivery)**	**(≥ 37 weeks at delivery)**			
Neonatal abstinence syndrome hospitalizations, % (***N***)	27% (460)	73% (1242)			
Mean length of stay in hospital (days)					
Acute inpatient care**	20.0 days	14.7 days	5.97 (791)		< 0.0001
Alternate level of care	0.1 days	0.1 days	−0.58 (1700)		0.5641
Total length of stay**	20.1 days	14.8 days	5.94 (790)		< 0.0001
Special Care Unit admission, % (***N***) **	89% (411)	64% (793)		105.45(1)	< 0.0001
Discharge disposition, % (***N***)					
Home with additional support*	20% (94)	14% (175)		10.15 (1)	0.0014
Home without additional support**	61% (280)	74% (920)		28.14 (1)	< 0.0001
Transferred**	18% (84)	12% (144)		12.86 (1)	0.0003
Left against medical advice, absent, or did not return from pass	Suppr.	Suppr.			
Died in hospital	Suppr.	Suppr.			

*Suppr.*, data are suppressed in accordance with CIHI’s privacy policy

*p < 0.05

**p < 0.001

## Discussion

This study found an increasing trend in the rate of NAS hospitalizations in Canada (excluding Quebec) between 2010 and 2020, and there appeared to be a slight dip in 2019. Opioid toxicity deaths also decreased briefly in 2019, which suggested that efforts to address the opioid crisis in Canada had been making gains prior to the COVID-19 pandemic (Public Health Agency of Canada, 2020). A seasonal pattern was identified, where the rates of NAS were lowest in quarter 2 (April to June) and highest in quarter 4 and quarter 1 (October to March). For all NAS hospitalizations, there was no statistically significant difference in mean length of stay in acute inpatient care during the observed time period, while there was a significant difference in mean length of stay in alternate level of care. Approximately 71% of NAS hospitalizations were admitted to the Special Care Unit and most discharges (72%) resulted in the newborn being sent home without additional support. Finally, pre-term births differed from term births in that they had longer length of hospital stays and increased rates of Special Care Unit admissions.

An overall increasing yearly trend in NAS hospitalizations is consistent with previous literature (Canadian Institute for Health Information, 2018; Lisonkova, 2019; Filteau, 2018; Turner, 2015) and mimics other increasing harms due to the opioid crisis in Canada (Public Health Agency of Canada, 2021a). However, it is unknown whether the incidence and length of stay trends identified are due to actual changes, or due to increased awareness and detection of NAS by healthcare professionals and changes in pharmacological and non-pharmacological practices. In addition, NAS scoring tools (i.e., Finnegan Neonatal Abstinence Scoring Tool) have limitations and modified or unvalidated versions are often used which can impact detection. NAS scoring tools may also not be appropriate for pre-term infants (Perinatal Services BC, 2020). Furthermore, in recent years in Canada, there has also been an increase in the use of opioid agonist therapy, such as methadone and buprenorphine, to treat opioid use disorders (IQVIA, 2021). While opioid agonist therapies are standard for the treatment of opioid use disorder during pregnancy, both methadone and buprenorphine may still contribute to a NAS diagnosis (Jones et al., 2010). However, preliminary research has shown in comparison to methadone, buprenorphine exposure in utero is associated with significantly shorter hospital stays and treatment for NAS (Bivin et al., 2019).

As far as we are aware, the seasonal trend identified in this study has not previously been reported in Canada. A seasonal trend may be explained by seasonality of other opioid-related harms and at what point during pregnancy a fetus is most vulnerable to substances. For instance, evidence suggests that some opioid-related harms, such as opioid-related poisoning hospitalizations, are lowest during the first and fourth quarter of each year (Public Health Agency of Canada, 2021a). It has also been noted that the risk of NAS is thought to be highest when a pregnant person uses opioids during their third trimester (Wen et al., 2021; Källén and Reis, 2016). Therefore, one possible explanation for why this study identified a seasonal dip in NAS hospitalizations between April and June is that a pregnant person’s third trimester, when they are most at risk of NAS, is aligning with when other opioid-related harms are consistently lowest. More research is required however to fully examine the cause of this trend.

While this study found that approximately 71% of NAS hospitalizations required a Special Care Unit admission, in contrast the Canadian Perinatal Surveillance System reports that approximately 12% of all newborn hospitalizations are admitted to a Special Care Unit in a given year (Public Health Agency of Canada, 2021b). Similarly, this study found that total length of stay was approximately 15 days for neonates hospitalized with NAS, while the Canadian Perinatal Surveillance System finds that the mean total length of stay in hospital for all newborns born between 2008 and 2019 was 2 days (Public Health Agency of Canada, 2021b). Previous literature indicates that newborns with NAS are more likely to be born pre-term (Stover and Davis, 2015). Additionally, the Canadian Perinatal Surveillance System indicates that among all hospitalizations for pre-term births from April 2019 to March 2021, approximately 63% were admitted to a Special Care Unit and the mean total length of stay was 11 days (Public Health Agency of Canada, 2021b). Therefore, the utilization patterns shown in this study are likely a result of both NAS status and pre-term birth status. This contrast further elucidates the increased hospital resources required to care for newborns hospitalized with NAS. Our study also did not detect a difference in hospitalization characteristics and outcomes during the COVID-19 pandemic, although further research examining more recent data years is warranted as impacts could be lagged. Moreover, this study did not identify a statistically significant increase in mean acute length of stay in hospital across Canada, in contrast to one study that found four Canadian provinces experienced a large increase between 2003 and 2014 (Filteau et al., 2018). Research for specific jurisdictions is required to determine whether there is variation across provinces and territories. Longer hospital stays, regionally and over time, may be due to increasing severity of NAS, variability in management practices for both the newborn and birthing parent, or better detection of the syndrome among high-risk groups (e.g., premature newborns) where the newborn may have lower severity of NAS, but longer stays due to other comorbidities (Haas, 2019).

### Future directions

Currently, the ICD-10-CA code used to identify NAS does not include information on type of opioid exposure. To remedy this, more specific diagnostic ICD-10-CA codes are needed to identify substance-specific causes for NAS. This would aid in determining what interventions are appropriate to reduce the burden of NAS. Additional research would also be beneficial to understand the long-term impacts of NAS, how to best support those affected, and how to adequately educate people who are pregnant to prevent cases of NAS. Finally, enhancing existing data systems to include more sociodemographic information on those who are admitted to hospital is warranted, such as information regarding ethnicity and socioeconomic status, in order to facilitate analysis of NAS rates by social determinants of health. This would help to identify and establish targeted, preventive approaches.

### Limitations

This study is not able to determine the source of exposure causing NAS, and therefore it could be due to various forms of use such as treatments for opioid use disorders, chronic opioid analgesic use, or unregulated opioid use. We were not able to identify any studies assessing the validity of the ICD-10-CA code P96.1 in Canada at the time of this study. However, a large population-based cohort of Medicaid participants in the United States found that the use of P96.1 in their hospital administrative data had a high positive predictive value of 98.2% (95% confidence interval: 95.4–99.2) for identifying cases of clinically diagnosed NAS (Maalouf et al., 2019). In addition, this study represents the number of hospitalizations for NAS and does not reflect the number of infants experiencing NAS. It is possible an infant may have been hospitalized twice in their first 28 days of life, or transferred to another facility for additional care and therefore would be captured more than once*.* Newborns who were not admitted to a hospital within the first 28 days of their birth could also not be included in this analysis. Furthermore, cases of NAS may go undiagnosed due to milder symptoms undetected by a caregiver or healthcare professional, delayed onset of symptoms post-delivery, and undisclosed use of substances by the pregnant parent. Due to these factors, the data presented in this study cannot be used to estimate a true prevalence of NAS in Canada.

## Conclusion

There were almost 17,000 neonatal abstinence syndrome hospitalizations between 2010 and 2020 in Canada (excluding Quebec) and both the number and rates of NAS hospitalizations have been increasing. Infants born with NAS, on average, require high healthcare resource utilization, as evidenced by their lengthy hospital stays and high proportion of Special Care Unit admissions. These findings highlight the need for prevention of substance-related harms among people who are pregnant and their newborns through support and additional harm reduction strategies. This study can help inform the need to establish and increase accessibility of opioid agonist therapy programs specifically designed for people who are pregnant, as well as the need for medical follow-up care and social services post discharge to manage long-term outcomes (McQueen and Murphy-Oikonen, 2016).

## Contributions to knowledge

What does this study add to existing knowledge?

- Our findings add to existing research documenting the increase in substance-related harms in Canada.
- We found that there was an 80% increase in the rate of neonatal abstinence syndrome hospitalizations in Canada (excluding Quebec) from 2010 to 2020. Newborns hospitalized with neonatal abstinence syndrome used a high level of healthcare in terms of length of stay in acute inpatient care and Special Care Unit admissions.
- This was the first Canadian study, to our knowledge, to find a statistically significant seasonal pattern in the rate of neonatal abstinence hospitalizations.

What are the key implications for public health interventions, practice, or policy?

- Knowing to what extent there has been an increase in neonatal abstinence syndrome hospitalizations can help inform additional prevention and harm reduction activities.
- Data systems could be strengthened to capture additional sociodemographic information in order to understand and support populations who are most at risk.

## Declarations

### Disclaimer

Parts of this material are based on data and information compiled and provided by CIHI. However, the analyses, conclusions, opinions, and statements expressed herein are those of the authors, and not necessarily those of CIHI.
